# Bell Peppers (*Capsicum annum* L.) Losses and Wastes: Source for Food and Pharmaceutical Applications

**DOI:** 10.3390/molecules26175341

**Published:** 2021-09-02

**Authors:** Luis Miguel Anaya-Esparza, Zuamí Villagrán-de la Mora, Olga Vázquez-Paulino, Felipe Ascencio, Angélica Villarruel-López

**Affiliations:** 1 Departamento de Ciencias Pecuarias y Agrícolas, Centro Universitario de Los Altos, Universidad de Guada-lajara, Av. Rafael Casillas Aceves 1200, Tepatitlán de Morelos 47620, Mexico; luis.aesparza@academicos.udg.mx; 2 Departamento de Ciencias de la Salud, Centro Universitario de Los Altos, Universidad de Guadalajara, Av. Rafael Casillas Aceves 1200, Tepatitlán de Morelos 47620, Mexico; blanca.villagran@academicos.udg.mx; 3 Departamento de Farmacobiología, Centro Universitario de Ciencias Exactas e Ingenierías, Universidad de Guadalajara, Blvd. Gral. Marcelino García Barragán 1421, Olímpica, Guadalajara 44430, Mexico; olga.vazquez@academicos.udg.mx; 4 Centro de Investigaciones Biológicas del Noroeste (CIBNOR), Av. Instituto Politécnico Nacional 195, Playa Palo de Santa Rita Sur, La Paz 23096, BCS, Mexico

**Keywords:** food revalorization, bioactive compounds, biological activities, health benefits

## Abstract

Currently, the high added-value compounds contained in plant by-products and wastes offer a wide spectrum of opportunities for their reuse and valorization, contributing to the circular economy. The bell pepper (*Capsicum annum* L.) is an exotic vegetable with high nutritional value that, after processing, leaves wastes (peel, seeds, and leaves) that represent desirable raw material for obtaining phytochemical compounds. This review summarizes and discusses the relevant information on the phytochemical profile of bell peppers and their related biological properties as an alternative to revalorize losses and wastes from bell peppers for their application in the food and pharmaceutical industries. Bell pepper fruits, seeds, and leaves contain bioactive compounds (phenols, flavonoids, carotenoids, tocopherol, and pectic polysaccharides) that exhibit antioxidant, antibacterial, antifungal, immunosuppressive and immunostimulant properties, and antidiabetic, antitumoral and neuroprotective activities, and have a potential use as functional food additives. In this context, the revalorization of food waste is positioned as a technological and innovative research area with beneficial effects for the population, the economy, and the environment. Further studies are required to guarantee the safety use of these compounds and to understand their mechanisms of action.

## 1. Introduction

Despite the nutritional contribution of food (regardless of its plant or animal origin), much of it will not be consumed by humans or animals and will be discarded as waste [1]. Thus, the prevention of food loss and waste promotes a favorable impact on the environment and food security of the world population, contributing to economic development [2]. Besides prevention, the revalorization of these food wastes is a technologically viable strategy, using them as bioactive ingredients to generate new, potentially functional or nutraceutical products [3]. Worldwide, food losses and waste mainly occur in fresh fruits and vegetables (>40%), and are mainly associated with poor handling and storage during post-harvest [2,3,4], which can be a raw source of bioactive compounds.

The *Capsicum* genus belongs to the *Solanaceae* family, *Solanoicleae* subfamily, Solaneae tribe. Chili (*Capsicum*), along with corn, beans, and squash, is one of the oldest cultivated plants in America [4]. There are five commercially cultivated species of chili (*C*. *chinense, C*. *annuum*, *C*. *pubescens*, *C*. *baccatum,* and *C*. *frutescens*) and around 25 wild and semi-cultivated species [5]. Peppers (*C*. *annuum* L.) are classified as hot or sweet; they are grown in subtropical climate regions throughout the world, including Mexico [6,7].

The production of bell peppers has increased considerably in recent years; however, the annual losses of this crop are estimated to be 40% [8]. Bell peppers can be of different colors (red, green, orange, and yellow) depending on their ripening stages and capacity to synthesize chlorophylls or carotenoids. Besides having an exotic flavor, bell peppers are an important source of vitamins (provitamin A, E, and C) and various bioactive compounds (phenolic compounds and carotenoids) that are beneficial for the health of consumers [9]. Additionally, scientific evidence shows that bioactive compounds extracted from bell peppers have anti-inflammatory, antidiabetic, antimicrobial, and immunomodulatory effects, among others [10,11,12]. This review summarizes and discusses relevant information on the phytochemical profile of bell pepper fruits and their related biological properties as an alternative to revalorize losses and wastes from bell peppers for food, cosmetic and pharmaceutical applications.

## 2. Potential Food Losses and Wastes to Contribute to a Circular Economy

Faced with the reduction in susceptible areas for agricultural and livestock activity due to desertification processes or the appearance of pests and diseases that decrease agricultural yield, it is necessary to change the paradigms of how to project and make the agricultural industry more efficient. This implies the establishment of ecological, biotechnological, ethical, and social strategies to optimize the use of natural resources [1], leading to bioeconomic sustainability schemes throughout the food production chain [2].

In this regard, Brunori [3] “identifies the goal of the bioeconomy as the capacity to mobilize science to obtain high biovalue returns from low-cost living matter, for example organic waste, and includes the value of non-market goods associated with agriculture and food”. Derived from this approach, a new industry arises, the biorefinery, which is based on the idea of a stepwise treatment of biomass starting from the extraction of the highest added-value components down to energy production, in such a way as to make the most out of the limited biomass resource availability (cascading approach). The order of actions determines the range of products obtained. By producing multiple products, biorefineries take advantage of the various biomass components and their intermediates, therefore maximizing the value derived from the biomass feedstock. In this sense, the biorefinery concept is often seen to improve the economic performance of the bioeconomy [4,5]. A relevant aspect of the biorefinery is its potential to appraise waste food as a renewable raw material to retrieve biomaterials and generate bioenergy through biotech strategies. This holistic perspective integrates remediation and resource recovery and can be implemented using different technologies [6].

From the bioeconomic perspective, it is fundamental to accomplish several aims simultaneously: (i) efficacious management of resources; (ii) biodiversity and soil conservation; (iii) assurement of ecosystemic services; (iv) appraisal of food losses and waste; v) bioenergy generation through biorefineries [1]. Smith et al. [7] reviewed the need to change land management practices and food production schemes to face the global land challenges of climate change mitigation and adaptation, combatting land deterioration and drought, and assuring food security. The practices considered by Smith et al. [7] can be catergorized into those that rely on the land, value chain, and risk management. Dietary change and waste reduction practices can provide enormous benefits for mitigation actions to reduce the negative impacts of climate change. In addition, reducing food waste and losses can relieve pressure on the global freshwater resource, thereby aiding adaptation [7].

A set of processes relevant for the bioeconomy is represented by technologies that allow for biomass valorization from waste. This also highlights the circular economy approach. Biowastes can come from several sources, including agriculture and food wastes, biowastes from other bio-based processes and biowastes from non-bio-based products, such as urban organic wastes and wastewater. The use of biowastes is still hindered by a lack of information about biowaste availability and a lack of integrated modular approaches and technology development, as well by legislative constraints [8]. According to the European Commission [9], some of the main areas of interest to ensure a circular economy include:

**(i)**  The cascading approach to use bio-based resources, including food waste.
**(ii)**  The potential for innovation in new bio-based materials, chemicals and processes contributing to the circular economy.
**(iii)**  The recycling of wood packaging and separate collection of biowaste.

Additionally, due to the growing population and changing diet habits, the production and processing of horticultural crops have increased significantly to fulfill the increasing demands. However, this increment in food processing has raised economic and ecological issues because of the generation of enormous amounts of food losses and waste. Estimated food losses and waste, published by FAO, indicate that losses and waste in fruits and vegetables are the highest among all types of foods and may reach up to 60%. The processing operations of a whole commodity group (fruits and greens) can generate up to 30% of food wastes. The important point is that food losses and waste are valuable sources of biologically active components, such as antioxidants, complex soluble polysaccharides, vitamins, enzymes, and fatty acids, among other bio-reagents that can be employed in several industries, including the food, health, medicine and pharmaceutical industries. In addition, all these bio-reagents have been reported to be beneficial for both human and animal health because of their antioxidative, anti-inflammatory, and immunomodulatory, and several other biological activities (see below) [10]. Thus, the employment of food losses and waste to obtain various bioactive components is essential for sustainable development [11].

Fruit and vegetable losses and waste constitute an economic and ecological important source for isolating of bioactive compounds that have to be valorized instead of terminated as waste in landfills. From the economic perspective, besides using them as animal feed or biogas production, there is a surplus of alternatives that can be used in the food, cosmetic and pharmaceutical industries due to their bioactive compounds. On the ecological side, the impact of fruit and vegetable losses and waste on environmental and human health has relevant importance. Thus, for their successful appraisal, an interdisciplinary approach must be established and correlated with effective citizen awareness campaigns to reduce food waste in compliance with national and international regulations to ensure the safety and agreement of any new product brought into the market [12].

The concept of the circular economy was conceived by fostering a change from a linear economy outline of “take-make-use-dispose” to a circular scheme, employing reuse, sharing, repair, refurbishment, remanufacturing, and recycling to create a closed-loop framework to minimize the use of natural resource inputs and the generation of waste, contaminants, and carbon emissions. High-added-value molecules contained in food losses and waste offer a wide spectrum of possibilities for appraisal and reuse, as envisaged by the circular economy [13]. The versatility of and interest in applying the principles of the circular bioeconomy make the biorefineries one of the valorization strategies for agro-food industrial residues that can positively impact the environment. In this context, one of the most popular crops worldwide is *Capsicum annum*, particularly bell peppers, which are considered as an exotic fruit with a high nutritional value [14]; nonetheless, this crop has bioactive compounds that have exhibited potential for industrial and pharmaceutical uses [15,16], as discussed below.

## 3. Description of Bell Peppers

*Capsicum annuum* is an annual or biennial herbaceous plant. Its lifecycle comprises four phases: seedling, vegetation, flowering and fruiting [17]. The bell pepper fruit is large and fleshy, quadrangular, of variable size (7–16 cm long/6–11 cm width) and weight (from 100 to 500 g). The consumers appreciate them for their exotic colors (green, red, yellow, and orange), flavor, and texture. Furthermore, they are usually consumed fresh, but are also frequently used to enhance food dishes or other food products [18,19]. Moreover, they can be processed in commercial products, such as sauces, puree, and powders, among others [20]. On the other hand, bell pepper fruits could be a good source of human nutrition and health by providing energy and bioactive compounds [21], as discussed below.

According to the literature, bell peppers have high levels of water and carbohydrates with a low protein and fat content, which makes them a low-calorie food; they also have an adequate content of dietary fiber to be considered a food that is high in fiber, which has important implications for the health and nutrition levels of consumers (Table 1). Additionally, bell peppers contain some nutritionally important compounds such as vitamins (B, A, D, C, E, and K) and minerals (potassium, sodium, magnesium, calcium, and phosphorus) [22]. In this sense, the frequent consumption of bell peppers provides essential nutrients for human health [23,24,25]. For example, fresh bell pepper consumption (100 g) provides the total ascorbic acid recommended daily intake [26]. On the other hand, the nutritional content of bell peppers depends directly on the color of the fruit, growing conditions, and postharvest processing, among other factors [27].

Additionally, the total pepper production has increased significantly (25%) in recent years (from 2006 to 2016) [14], and it is one of the most commercially cultivated vegetable crops worldwide [29]. For example, in Mexico, the total production of bell peppers was 676,216 tons during 2019 [17]. Therefore, the recovery of bell pepper phytochemicals offers a viable strategy to obtain bioactive compounds, which could be used as natural ingredients for the food and pharmaceutical industries, as an alternative to replacing synthetic compounds and also in the revalorization of a plant’s wastes and losses [16].

## 4. Phytochemicals Present in Bell Peppers

The bell pepper is highly consumed worldwide due to its exotic colors (green, red, yellow, orange, and purple), flavor, and nutritional value [30]. However, after processing bell pepper products, waste remains (seeds, peel, stem, and leaves), representing desirable raw material to obtain phytochemical compounds [20]. They contain diverse bioactive compounds with interesting biological activities (in vivo and in vitro) and applications [31]. Bioactive compounds are defined as “inherent non-nutrient constituents of food plants with anticipated health-promoting/beneficial and/or toxic effects when ingested” [32]. Their concentration depends on the fruit part (seeds, peel, and pulp), the variety or cultivar, postharvest conditions such as maturity stage, storage conditions, and processing practices [16]. In general, there is an extensive bioactive compounds screening in bell pepper fruits (qualitative or quantitative), and the identified phytochemicals include phenolic, flavonoids, and carotenoids. All these compounds show great potential for use in pharmaceutical and food industries, as discussed below.

### 4.1. Phenols and Flavonoids

Phenols and flavonoids are one of the most abundant phytochemicals in fruits and vegetables. They have shown antioxidant effects and exhibited potential health benefits for the human body [33]. In this context, bell pepper fruits are an excellent source of phenolic acids and flavonoids [34], as listed in Table 2.

In general, the concentration of polyphenol compounds in bell peppers varies with variety and color, ranging from 5.59 to 52.65 mg of gallic acid equivalent per gram of edible portion [22,23,24]. The main phenolic compounds found in green bell peppers include myricetin (658 µg/g), pyrogallol (572 µg/g), chlorogenic acid (290 µg/g), catechol (279 µg/g), protocatechuic acid (116 µg/g), caffeic acid (108 µg/g), ellagic acid (106 µg/g), and gallic acid (89 µg/g) [23,27,35]. In red bell pepper, pyrogallol (757 µg/g), P-OH benzoic acid (395 µg/g), myricetin (244 µg/g), chlorogenic acid (221 µg/g), ellagic acid (172 µg/g), gallic acid (115 µg/g), and benzoic acid (111 µg/g) [23,26,27,35] were found; while in yellow bell pepper, pyrogallol (2175 µg/g), catechol (225 µg/g), ellagic acid, benzoic acid (173 µg/g), myricetin (151 µg/g), ellagic acid (144 µg/g), chlorogenic acid (136 µg/g), and P-OH benzoic acid (123 µg/g) [23,27] were found. Furthermore, it has been reported that orange bell pepper contains gallic acid (900 µg/g), chlorogenic acid (117 µg/g), myricetin (100 µg/g), resveratrol (89 µg/g), caffeic (38 µg/g), ferulic (13.45 µg/g) and p-coumaric acids (13.45 µg/g) [29,30,35]. Additionally, the presence of gallic acid has been reported in purple (1200 µg/g) and dark violet (1150 µg/g) bell peppers [35]. These compounds exert potent antioxidant activity against reactive oxygen species and reactive nitrogen species [36].

Similar to phenolic acids, the flavonoid content depends on the variety and color of bell peppers, ranging from 2.1 to 41 mg of quercetin equivalent per gram of edible portion [23,24,34]. The main flavonoids reported in green bell pepper were hesperidin (1065 µg/g), quercetin (394 µg/g), catechin (295 µg/g), naringin (275 µg/g), luteolin 7-glucose (181 µg/g), Apig. 6-rhamnose 8-glucose (170 µg/g), and Apig. 6-arbinose 8-galactose (151 µg/g) [29,30,31,34,35]; while in red bell pepper, hesperidin (1513 µg/g), catechin (793 µg/g), luteolin 7-glucose (413 µg/g), Apig. 6-rhamnose 8-glucose (314 µg/g), rutin (290 µg/g), quercetin (241 µg/g), and Apig. 6-arbinose 8-galactose (156 µg/g) [22,23,24,27] were reported. On the other hand, in the yellow bell pepper, catechin (745 µg/g), hesperidin (213 µg/g), naringin (190 µg/g), quercetin (102 µg/g), luteolin (95 µg/g), luteolin 7-glucose (92 µg/g), and Apig. 6-arbinose 8-glucose (77 µg/g) [29,30,31,34,35] were reported. Moreover, the presence of quercetin (92 µg/g) and luteolin (56 µg/g) has been reported in orange bell pepper fruit [30]. Additionally, other colored bell peppers, such as purple and dark violet, have been reported to contain catechin (3.67 and 15.54 µg/g, respectively) and quercetin (38.04 and 117.58 µg/g) [35]. These compounds have shown antioxidant, anti-inflammatory, and antidiabetic effects [37,38].

Evidence indicates that bell pepper fruits are rich in phenolic compounds and flavonoids that may improve the human health status [22,23]; moreover, there is an association between uptake diets rich in phytochemicals and the risk reduction of chronic non-communicable diseases such as diabetes, osteoporosis, and cancer [16]. In most cases, the biological effects of polyphenols and flavonoids have been attributable to their antioxidant capacity, which can mitigate oxidative stress [27]. Thus, the regular consumption of bell peppers can improve human health and prevent degenerative diseases [38,39].

### 4.2. Carotenoids

Carotenoids are natural and lipophilic pigments found in colored fruits and vegetables [16]. They are isoprenoid compounds and are responsible for the exotic color in red, yellow, and orange bell peppers [40]. In general, carotenoids have antioxidant activity and exhibit essential functions in human nutrition and health [41]. Table 3 shows the carotenoid content of bell pepper.

In general, the concentration of carotenoids in bell pepper depends on their color [26] and ripening state [44], where the highest concentration was reported in red bell pepper (7137–8800 µg/g), followed by orange (5292 µg/g), yellow (2236.3–2834 µg/g), and green (1219–1513.5 µg/g) peppers [23,26,27,42]. The main carotenoids reported in green peppers include neoxanthin (190 µg/g), chlorophyll (150 µg/g), lutein (76 µg/g), zeaxanthin (35 µg/g), and capsanthin (16 µg/g) [22,24,43,44], while, in red bell peppers, the most reported carotenoids were 5,6,-epoxide capsanthin (513 µg/g), lycopene (322 µg/g), capsanthin (178 µg/g), cucurbitaxanthin (81 µg/g), and Zeaxanthin (70 µg/g) [22,27,43,44,46,47]. In yellow bell peppers, the most common were lutein (115 µg/g), chlorophyll (61 µg/g), zeaxanthin (48 µg/g), capsanthin (45 µg/g), and α-carotene (21 µg/g) [22,24,34,44,45]. Moreover, the main carotenoids reported in orange bell peppers were zeaxanthin (191 µg/g), β-carotene (56 µg/g), lutein (45 µg/g), capsanthin (45 µg/g), and β-cryptoxanthin (19 µg/g) [22,27,34,44,45]. Carotenoids are excellent antioxidant compounds with several human health benefits; their consumption may prevent coronary heart diseases and some types of cancer (gastrointestinal, lung, prostate, and breast), and can reduce the risk of age-related macular degeneration, as well as having a beneficial effect on cognitive function [46]. However, specific carotenoids may provide specific health benefits; for example, α- carotene, β-carotene, and β- cryptoxanthin are provitamin A compounds [48]. Moreover, β-carotene has positive effects on cognitive functions, while lutein and zeaxanthin provide eye protection [48]. Furthermore, lycopene exhibited potent antioxidant activity and may reduce cholesterol in animals [48]. According to these data, the regular consumption of bell pepper fruits may improve human health.

### 4.3. Other Phytochemicals Identified in Bell Peppers Fruits, Seeds, and Leaves

Other bioactive compounds such as colneleic acid, capsoside A, gingerglycolipid A, and blumenol C glucoside have been qualitatively identified in red and yellow bell pepper fruits [36]. Stuliff et al. [31] reported 57 glycerolipids and glycerophospholipids, two sphingomyelin compounds and ceramides in green, yellow, and red bell peppers. These compounds could exert interesting biological activities. Additionally, some glycosides, saponins, and alkaloids have been identified in yellow, red, and green bell peppers [23]. Furthermore, Adami et al. [49] reported that green sweet pepper contains pectic polysaccharides composed of uronic acids (67%), with minor amounts of rhamnose (1.6%), arabinose (6.4%), xylose (0.3%), galactose (6.7%), and glucose (4.4%) and reported that these compounds exhibited antineoplastic effects against mammary tumor cells.

Blanco-Rios et al. [27] stated that green, red, orange, and yellow bell peppers contained α-tocopherol (0.98, 3.65, 1.92, and 1.23 mg/g, respectively) and mentioned that this compound is important to human health due to its strong antioxidant activity. Furthermore, González-Zamora et al. [50] reported that bell peppers contain homodihydrocapsaicin (0.41 mg/g).

On the other hand, Silva et al. [51] reported that seeds of bell peppers contain sterols, and triterpens such as botulin (161 mg/kg), campesterol (54.1 mg/kg), stigmasterol (9.3 mg/kg), and β-sistosterol (67.4 mg/kg), as well as fatty acids such as myristic (236 mg/kg), pentadecylic (157 mg/kg), palmitoleic (764 mg/kg), palmitic (15,041 mg/kg), margaric (22,463 mg/kg), stearic (11,693 mg/kg), and arachidic (689 mg/kg). These compounds exert antioxidant activity and acethylcholinesterase-inhibitory effects.

Additionally, Dias-Games et al. [52] isolated a Hevein-like peptide from the leaves of bell pepper crops. These compounds showed antibacterial and antifungal activities and exhibited great potential for biotechnological use [53].

## 5. Biological Activities of Bell Pepper Extracts

As discussed in the preceding sections, bell pepper fruits, seeds, and leaves contain bioactive compounds (phenolic, flavonoid, carotenoids, tocopherol, and pectic polysaccharides), which are associated with different biological activities for diverse applications, as described below.

### 5.1. Antioxidant Activity

Bell peppers are characterized by their exotic colors and nutritional value, but also provide significant amounts of phytochemicals such as phenolic, flavonoid, and carotenoids with a strong antioxidant capacity [54]. Table 4 lists various reports on the antioxidant effects of bell pepper fruit extracts (pulp, juice, and seeds), where the most studied models include DPPH, ABTS, and FRAP.

The antioxidant activity of any fruit or vegetable is determined by different bioactive compounds, showing different action mechanisms to inhibit radicals. Therefore, more than one method should be used to clarify the mode of action of each extract or compound from bell peppers. In this context, the antioxidant properties of bell peppers are an important parameter for establishing their health functionality.

Chávez-Mendoza et al. [54] reported that the antioxidant activity of ethanolic extract from grafted bell peppers depends on the cultivar, color, concentration, and type of bioactive compound. They demonstrated that red bell peppers exhibited higher antioxidant capacity (79.65%), followed by green (78%), orange (70%), and yellow (64.90%) peppers, using the DPPH test, which was associated with the phenolic and β-carotene content of each cultivar; this behavior, in antioxidant activity, was promoted by grafting [46]. Conversely, Guil-Guerrero et al. [45] reported that ethanolic extract from yellow bell pepper showed the highest antioxidant activity (80% by DPPH assay) compared to the orange (75%), red (50%), and green (40%). According to Navarro et al. [47], the content of antioxidant compounds depends on the harvest conditions and the ripening stage. On the other hand, ethanolic extracts from bell peppers exhibited similar inhibition values to oxidize the DPPH radical compared to commercial antioxidants (50 to 80%) [45]. This information supports the use of plant materials for a myriad of disease conditions.

Similarly, Abdalla et al. [23] reported that red, yellow, and green bell pepper extracts (IC_50_ from 1114 to 1832 µg/mL) exhibited higher antioxidant activity than commercial antioxidant BHT (IC_50_ of 145.23 µg/mL) using DPPH assay in a fruit-color-dependent manner; where the highest antioxidant activity was obtained by yellow bell pepper extract (IC_50_ of 3267 µg/mL). Additionally, the same extracts showed a higher reducing power activity (IC_50_ from 451 to 3813 µg/mL by FRAP assay) in comparison with ascorbic acid (IC_50_ of 196 µg/mL), where the highest activity was observed in red bell pepper extracts (IC_50_ of 3813 µg/mL). These results were associated with the bioactive compounds, particularly with the flavonoid content in bell peppers, which can reduce oxidative stress.

Park et al. [55] investigated the antioxidant activity of methanolic extracts from four different-colored bell peppers by different methods (ABTS, DPPH, and SOD-like activity). They informed that extracts from orange bell peppers showed the highest antioxidant activity by ABTS test (880 µmol TE/g) compared to the red (800 µmol TE/g), yellow (790 µmol TE/g), and green (630 µmol TE/g) bell peppers; in contrast, the green bell pepper exhibited the highest antioxidant activity by DPPH assay (1153 µg/mL) compared to red (882 µg/mL), yellow (811 µg/mL), and orange (694 µg/mL) bell peppers. Furthermore, the green bell pepper showed the highest SOD-like activity (IC_50_ = 1472 µg/mL) compared to the yellow (IC_50_ = 1676 µg/mL), red (IC_50_ = 1826 µg/mL), and orange (IC_50_ = 1893 µg/mL) bell peppers. The authors argued that differences in results in colored bell peppers are attributable to the mode of action of each bioactive compound present in the extract and their ability to reduce or inhibit oxidative stress, which is dependent on the color of the fruit. Additionally, the extracts from the four bell peppers exhibited a protective effect against H_2_O_2_- and HNE-induced DNA damage at low concentrations (1 µg/mL), preventing cellular damage at physiological levels. These compounds could form a reversible complex that may act as a desmutagenic molecule or interceptor, inhibiting genotoxicity [55].

Qiao et al. [59] informed that the antioxidant activity of green, red, and yellow bell pepper extracts could be classified as FRAP > DPPH > ABTS. Nonetheless, the highest antioxidant activity was observed in yellow bell pepper in DPPH and ABTS methods, while, for FRAP, this was observed in the green bell pepper extract. Moreover, these extracts showed anti-inflammatory properties.

Thupairo et al. [22] investigated the effect of solvent extraction (hexane, ethyl acetate, and aqueous-ethanol) on the antioxidant properties (DPPH, FRAP, and ORAC tests) of colored bell peppers (green, red, orange, and yellow). They showed that the antioxidant activity of all fruits was dependent on solvent extraction, where the highest activity was obtained using aqueous-ethanol extraction (2- to 8-, and 300 to 80-fold higher than ethyl acetate and hexane extraction, respectively). According to the authors, the polarity of ethanol promotes the extraction of phenolic compounds, which exhibited potent antioxidant activity. On the other hand, the highest antioxidant activity was obtained in green bell pepper by all three investigated methods (25.15, 30.15, and 61.96 µmol TE/g, respectively), followed by the red (23.79, 28.12, and 54.81 µmol TE/g, respectively), orange (22.20, 25.20, and 51.12 µmol TE/g, respectively), and yellow (18.63, 19.87, and 51.65 µmol TE/g, respectively) peppers. This behavior was attributed to the higher ascorbic acid and phenolic content of green and red bell peppers, especially by *p*-coumaric and ferulic acids. Moreover, these extracts exhibited cholinesterase inhibitory effects. Additionally, Zhuang et al. [26] reported that red bell pepper showed antioxidant activity (by DPPH and FRAP assays) that may prevent lipid peroxidation. Similar trends were reported by Prabakaran et al. [63], who informed that ethanolic red bell pepper extract showed higher antioxidant activity than chloroform and ethyl acetate extracts in both DPPH and FRAP tests; however, their effects were dose-dependent. Furthermore, Bae et al. [65] reported that a higher DPPH scavenging activity was detected in the hexane extracts from bell pepper fruits, whereas the reducing power (FRAP) was detected in ethyl acetate and acetone extracts.

Blanco-Rios et al. [27] showed that phenolic and oily fractions of colored (red, green, orange, and yellow) bell peppers exhibited antioxidant activities using ABTS and DPPH tests. They demonstrated that the highest antioxidant properties were detected in phenolic fractions compared to oily fractions (associated with carotenoids and tocopherol content) in both studied methods. Ilić et al. [61] reported that lipophilic fraction from orange bell pepper (Sympathy cv.) exhibited higher antioxidant activity using the ABTS test (5.20 µmol TE/g) than red (4.05 µmol TE/g, Selika cv.), and yellow (3.33 µmol TE/g, Dynamo cv.) bell peppers. These results evidenced the potential of bell peppers to improve human health.

Norafida and Aminah [56] reported that blanching treatments (microwave > hot water > steam) influenced the antioxidant properties of frozen green bell peppers. Similar trends were reported in cooked bell peppers by microwave and stir-frying without using water [64]. Moreover, it has been reported that sun-drying significantly increases the antioxidant properties (ABTS and DPPH tests) (55.64 µmol TE/g and 76%) compared to a fresh product (17.33 µmol TE/g and 48%) using the ABTS and DPPH tests [62]. According to Shotorbani et al. [58], during thermal treatments, the generation of different compounds with different degrees of antioxidant activity can occur, promoting higher antioxidant properties. The antioxidant activity of fresh bell pepper (regardless of cultivar) significantly increased during ripening; therefore, consumption in the fresh state is recommended at the mature stage [35].

Furthermore, an increase in the antioxidant activity (reduction in DPPH radicals from 54 to 70%) of red bell peppers cv. “California Wonder”, by applying a nanostructured coating of chitosan functionalized with *Byrsonima crassiflora* extracts, has been reported by González-Saucedo et al. [42]. This behavior could be attributable to the elicitor properties of chitosan, as demonstrated by Garcia-Mier et al. [66]. They showed that the antioxidant activity of bell pepper fruits increased with the elicitor agents (jasmonic acid, hydrogen peroxide, and chitosan) during postharvest. In addition, the bell pepper juice from green, yellow, orange, and red fruits exhibited antioxidant activity using the DPPH and ABTS methods, which could make it a viable option for bell pepper consumption [60].

Additionally, it has been reported that seed extract from bell pepper exhibited higher antioxidant activity than pulp. For example, Sousa-Sora et al. [57] studied the antioxidant activity of bell pepper fruits and seeds using DPPH, ABTS, and FRAP methods. They informed that seed extracts exhibited the highest antioxidant capacity (11.32, 89.25, and 9.94 µmol TE/g, respectively) than the pulp extracts (2.28, 17.17, and 3.99 µmol TE/g, respectively), which were associated with the total phenolic content of seeds (409 mg GAE/g) and pulp (119 mg GAE/g). Similarly, Silva et al. [51] informed that bell pepper seed extracts exhibited antioxidant activity in a dose-dependent response (IC_25_ of 0.413 µg/mL by DPPH assay), whereas, at higher concentrations, a pro-oxidant effect was noticed. Moreover, the seed extract showed an effect against nitric oxide radicals (IC_25_ of 0.105 µg/mL).

According to the evidence, colored bell peppers (pulp, juice, and seeds) are a good source of antioxidant compounds important for dietary consideration because they can prevent the formation of free radicals that can be harmful to human cells. Nonetheless, they have a key role in protecting against diverse non-communicable diseases.

### 5.2. Antimicrobial Activity

Natural extracts from leaves, seeds, and fruits are commonly used as antimicrobial agents. Table 5 shows the antimicrobial properties of bell pepper extracts.

Dorantes et al. [67] investigated the antimicrobial properties of bell pepper extracts against some foodborne pathogenic bacteria and showed that extracts exhibited effectiveness regarding the inhibition of *Listeria monocytogenes* (inhibition zone of 12 mm), *Bacillus cereus* (inhibition zone of 11 mm), *Staphylococcus aureus* (inhibition zone of 7 mm), and *Salmonella tiphimurium* (inhibition zone of 5 mm), which was associated with the presence of m-coumaric and cinnamic acids in the extract. Similarly, it has been reported that methanolic extracts from red and yellow bell peppers showed antimicrobial activity against some pathogenic bacteria. Hu et al. [68] showed that a minimal inhibitory concentration of red and yellow bell pepper extracts to inhibit the microbial growth was seen in a strain-dependent response. In particular, the extracts from red bell pepper were more active against *B. cereus* (0.20 mg/mL), *Escherichia coli* (0.50 mg/mL), *S. aureus* (0.30 mg/mL), and *Pseudomonas aeruginosa* (0.60 mg/mL) than the yellow extracts (0.40, 0.50, 0.40, and 0.60 mg/mL, respectively). According to the authors, these differences may be attributed to the phytochemical profile nature of each extract. Nonetheless, both extracts from bell peppers induced cell-wall damage, as demonstrated by transmission electron microscopy studies.

Mokhtar et al. [73] reported that extract from a bell pepper rich in polyphenols showed antimicrobial effects against *S. aureus*, *L. monocytogenes*, *S. typhimurium*, *E. coli*, *B. subtilis*, and *P. mirabilis* in a strain- and dose-dependent response. The authors argue that the polyphenols can alter the cell wall of pathogens, leading to cell death; moreover, they showed that the growth of *Lactobacillus acidophilus* and *L. plantarum* were stimulated by the presence of polyphenols, and mentioned that these compounds may exert a prebiotic-like effect via modulation of the gut microbiota. Additionally, Careaga et al. [71] reported that extracts from bell peppers are effective in preventing the growth of *S. typhimurium* and *P. aeruginosa* and mentioned that the extract showed a bacteriostatic (0.3 mL/100 g of meat) or bactericidal (3 mL/100 g of meat) effect in a dose-dependent response. Similarly, Aljaloud et al. [72] showed that crude extract from bell peppers showed antimicrobial activity against *E. coli* O157H:7 in a dose-dependent manner. These extracts could be used as an antimicrobial agent to preserve beef meat [71,72].

On the other hand, Días-Games et al. [53] investigated the in vitro antimicrobial activity of bioactive compounds extracted from bell pepper leaves. They showed that the extract exhibited antimicrobial effects against *Clavibacter michiganensis* spp. *michiganensis, Ralstonia solanacearum, Pseudomonas syringae* pv. *tomato, Xanthomonas axonopodis* pv. *phaseoli, Erwinia carotovora* spp. *carotovora* in a strain- and concentration-dependent response and mentioned that peptide compounds could be used as a biotechnological alternative for biological control of phytopathogens. Similarly, Dias-Games et al. [52] showed that the Hevein-like peptide extracted from bell pepper leaves showed antimicrobial properties against *Clavibacter michiganensis* spp. *michiganensis* (Gram-positive) and *Ralstonia solanacearum* (Gram-negative). These results were associated with the cationic character of the molecule due to their chitin-binding domain, which may induce changes in the bacterial cell wall, inducing cell death.

Additionally, it has been reported that ethanolic extracts from red bell peppers showed antifungal activity against *F. andiyasi* and *Cochliobolus* spp., associated with the phenolic content in the extract and their ability to alter the fungal cell wall, leading to cell death [69]. Moreover, the ethanolic extract of red bell peppers exhibited antifungal effects against *Colletotrichum gloeosporioides* in a dose-dependent manner, which could be used as an alternative to biocontrol of phytopathogens [70].

According to these data, the extracts from bell pepper fruits or leaves exhibited antibacterial and antifungal activities, which could potentially be used for food and pharmaceutical applications.

### 5.3. Immunomodulatory Activity

Table 6 lists reports on the various immunomodulatory effects exerted by bell pepper extracts (fruits and leaves), including immunosuppressive and immunostimulant effects.

Sancho et al. [74] reported that the nordihydrocapsiate (NHC), a compound extracted from sweet bell peppers, exhibited anti-inflammatory properties in a concentration dependent manner. They found that NCH inhibited the anti-CD3, anti-CD3 plus, and anti-CD28, promoting apoptosis (VR1-independent pathway) in primary T cells and inhibiting the NF- kBis by phosphorylation of MAPK p38. Similar trends were reported by Hazekawa et al. [75], who showed that the aqueous extract from bell pepper leaves strongly suppressed the stimulated T-cell activation and the iNOS and NF-κB expression levels in mouse spleen cells by Con-A in a dose-dependent manner, after 72 h of stimulation. Moreover, it has been reported that a pectic polysaccharide extracted from bell pepper fruit exhibited anti-inflammatory properties in a male BALB/c mice model [76]. According to Popov et al. [76], after 24 h of oral administration of pectic polysaccharides at concentrations of 40 to 100 mg/kg, the TNF-α release was decreased, with no impact on the number of monocytes and neutrophils.

Additionally, it has been reported that the administration of red and green bell pepper extracts stimulates the production of antibodies in murine spleen cells [77,78,79]. Goto et al. [79] reported that the red bell pepper extract enhanced the IgM production (by up to 350%) in a dose-dependent response (1.5 mg/mL) in mice spleen cells, due to an increase in DNA synthesis in B cells and CD138+ cells. Recently, Sarker et al. [77] showed that the red bell pepper extract promotes anti-keyhole limpet hemocyanin IgM and IgG antibodies production in a dose-dependent response (from 0.75 to 1.5 mg/mL). Furthermore, the red bell pepper extract promotes B cell differentiation to plasma cells, enhancing the production of antigen-specific antibodies. Additionally, Sarker et al. [78] reported that red bell pepper extracts promoted polyclonal IgM and IgG antibody production in murine spleen cells, which was associated with the phytochemical profile in the extract. According to the literature, the consumption of bell peppers improves the immune system, which was mainly attributed to the content of phenols, flavonoids, and carotenoids [68].

In general, the extracts from bell peppers (fruits and leaves) can modulate the immune response, exerting anti-inflammatory properties and promoting the production of antibodies. However, further studies are needed to understand the mechanism of action and potential applications.

### 5.4. Effect of Bell Pepper Extracts on Diverse Pathologies

Table 7 lists studies that reported the anti-hyperglycemic, cytotoxic, and neuroprotective effects of bell pepper extracts.

The antidiabetic properties of the bell pepper extract have been investigated in recent years. Park et al. [80] investigated the inhibitory effects of ethanolic extract from green bell pepper against α-glucosidase, and its insulin-like action, as an alternative to diabetes mellitus. They found that the extracts showed antidiabetic effects due to their ability to decrease intestinal glucose absorption. Moreover, bell pepper extracts exhibited insulin-like effects, which can induce 3T3-L1 cell differentiation. However, further studies are needed to understand the mechanism of action of each constituent in the extract that promotes antidiabetic properties. Conversely, Shukla et al. [84] showed that the methanolic extract from yellow bell pepper exhibited higher α-glucosidase-inhibitory effects than green or red bell pepper extracts, and prevents lipid peroxidation. These effects were attributed to the phytochemical composition of each extract and its antioxidant properties. The authors mentioned that bell pepper extracts might be helpful in diabetes mellitus management. Fuentes et al. [82] mentioned that red bell pepper extracts prevent the formation of protein islet amyloid polypeptide (IAPP) aggregates, indicating that the extracts exhibited potential antidiabetic properties and may slow disease progression.

Lahmood [83] showed that the mixture comprising a red bell pepper extract and virgin olive oil exhibited α-amylase- and α-glucosidase-inhibitory effects in a dose-dependent response (2 to 8 mL/kg body weight). Moreover, the mixture promotes a decrease in the serum glucose concentration and increases the insulin concentration in treated rats, which are key parameters in the management of diabetes mellitus type 2. Additionally, Nagasukeerthi et al. [81] reported that consuming 100 mL of bell pepper fruit juice twice a day reduces post-prandial blood glucose, systolic blood pressure, pulse pressure, and rate pressure compared to the control group. According to the authors, the antioxidant compounds of the extract may reduce the oxidate damage, increasing the sensitivity of pancreas β-cells to glucose.

According to these data, the extracts from bell pepper fruits exhibited antidiabetic effects, which were mainly associated with their phytochemicals and antioxidant properties and their ability to modulate the digestion of carbohydrates and improved insulin secretion and sensitivity.

Furthermore, the anticancer properties of bell pepper extracts have been studied in diverse cancer cell lines in recent years. E-Hamss et al. [85] investigated the antimutagenic properties of bell peppers *Drosophila melanogaster* larva (2-day-old). They reported that the bell pepper aqueous extract (10 *v*/*w*) significantly reduced the mutation induced by a promutagen agent (ethyl carbamate), associated with the ability of bioactive compounds in the extract (mainly carotenoids) and their ability to inhibit mutagens by modulating microsomal cytochrome. Moreover, the extracts did not show genotoxic effects against *D. melanogaster*.

Adami et al. [49] investigated the antineoplastic activity of green bell pepper pectic polysaccharides (GBPPP) against mammary tumor cells (in vivo and in vitro). They found that GBPPP reduces Ehrlich tumor development (from 28 to 54%) in a dose-dependent manner (50 to 150 mg/kg) compared to the control group, due to GBPPP’s ability to reduce the gene expression of Ehrlich tumors. Furthermore, the GBPPP showed inhibitory effects on tumor cell proliferation and viability against MCF-7, MDA-MB-231, and MDA-MB-436 cancer cell lines in a cell-line-dependent response. According to the authors, these effects were associated with increased intramural IL-6, which effectively stimulated angiogenesis.

Additionally, Hu et al. [68] investigated the effect of yellow and red bell pepper extracts against cancerous cells. They found that the extracts exhibited selective cytotoxic effects against the A549 cancer cell line at 125 µg/mL, whereas the extracts did not show any cytotoxic effect against NIH3T3 cells. Moreover, the red bell pepper extract is more active against the A549 cell line compared to the green extract. The anticancer effects were associated with the bioactive compounds (mainly carotenoids) that can induce apoptosis in cancer cell lines via the inactivation of NADH-oxidase. Jeong et al. [86] mentioned that the type and concentration of polyphenols strongly influenced the anticancer properties of bell pepper extracts. They found that the polyphenol mixture extracts effectively affected the viability of cancer cells (human gastric adenocarcinoma cells, A549 human lung carcinoma cells, and HeLa human cervical carcinoma cells) in a dose- and cell-type-dependent response (0.2 to 2 mg/L). These results were attributed to the strong antioxidant activity of the polyphenol mixture extract, which is composed of quercetin, luteolin, and their derivates; nonetheless, it has been reported that these compounds effectively inhibit the viability and proliferation of diverse human cancer cell lines.

According to the evidence, extracts or isolated compounds from bell peppers have the potential to treat different types of cancer. However, further studies are required for targeted antiproliferative activities.

On the other hand, it has been reported that bell pepper extracts exhibited beneficial effects in Alzheimer’s disease [87]. Ogunruku et al. [87] showed that the aqueous extracts from bell pepper can inhibit β-secretase in a dose-dependent manner, associated with the polyphenol compounds, and their antioxidant properties may cross the blood–brain barrier. Moreover, the extracts promote a decrease in peptide aggregation due to its ability to disaggregate fibrils, which could be a viable strategy in the prevention and management of Alzheimer’s disease. According to the literature, the dietary supplementation of phytochemicals exerts beneficial effects in the central nervous system due to its anti-aggregative and neuroprotective properties [88].

In general, phytochemicals present in bell pepper can also exert antihyperglycemic, cytotoxic, and neuroprotective effects. Further studies are still needed to validate these bioactivities.

### 5.5. Applications of Bioactive Compounds from Bell Peppers in Food Industry

In addition to the biological activities, natural compounds from bell pepper fruits have been used for diverse food industrial applications, as listed in Table 8.

Careaga et al. [71] reported that extracts from bell peppers are effective in extending the shelf life of raw beef meat (7 days stored at 7 °C) and prevent the growth of *Salmonella typhimurium* and *Pseudomona aeruginosa*, due to the bactericidal (3 mL/100 g of meat) effect of the extract. Likewise, the extracts from red bell peppers (5% *v*/*v*) effectively extend the shelf life or ground beef, associated with the ability of the natural extract to reduce microbial growth, particularly for *E. coli* O157H:7 [72].

On the other hand, bioactive compounds extracted from red bell peppers have been used as natural colorants of yogurt and isotonic drinks [20,89,90]. Mendes-Gomes et al. [89] added encapsulated bioactive compounds extracted from bell peppers to improve the color, sensory attributes, and nutritional value of yogurt. Furthermore, Šeregelj et al. [20] showed that the fortification of yogurt with natural colorants (from bell pepper wastes) improved its sensory attributes and positively affected the viability of lactic acid bacteria in yogurt during storage. Additionally, Lobo et al. [90] demonstrated that extracts from yellow bell peppers could be used as a natural pigment in isotonic drinks. In general, adding bioactive compounds from bell pepper wastes to foods as a functional ingredient is a technological and viable alternative to the valorization of food wastes [91].

According to the evidence, the phytochemicals present in bell pepper fruits could be used as antimicrobial agents for food preservation or as additives/bioactive ingredients to elaborate functional foods that may improve human health status.

## 6. Concluding Remarks

Sustainability in food production and consumption benefits all of the food chain and the environment. Efficient food production and supply chains, leading to less food loss, is only a part of sustainability. The other part is use, through the revalorization of waste and food losses. The valorization of food waste for edible purposes is an area of opportunity for researchers due to the significant content of the present bioactive compounds, which can be reused in numerous products, to which they will add value, thereby reducing the generation of waste food.

The revalorization of waste from fruits and vegetables, particularly bell pepper, mainly focuses on the use of bioactive compounds whose presence depends on the variety, color, growing conditions and degree of maturity during the harvest and post-harvest handling of the fruit, in addition to the source (seeds or leaves). In general, the bioactive compounds reported in bell pepper are phenolic compounds, flavonoids, carotenoids, tocopherol and pectic polysaccharides, which have antioxidant, antimicrobial, immunomodulatory activity. Besides their positive effects in the treatment of diseases such as diabetes mellitus, cancer and Alzheimer′s, bell pepper extracts can also be used as food additives (preservatives and colorants).

In this context, the revalorization of food waste is an area of opportunity for research and technological innovation, with beneficial effects for the population, the economy and the environment. However, most studies have been carried out in vitro, so in vivo investigations are also necessary to demonstrate the effectiveness of the aforementioned compounds, in addition to guaranteeing their safety and understanding their mechanisms of action.

## Figures and Tables

**Table 1:** Principal nutrient content by 100 g of bell pepper (*C. annuum* var. *annuum*).

Component	Value
Energy (Kcal/KJ)	26/111
Moisture (g)	92.2
Carbohydrates (g)	6.03
Dietary fiber (g)	2.1
Protein (g)	0.99
Total fat (g)	0.30
Ash (g)	0.47
**Vitamins**	
Niacin (mg)	0.979
Pyridoxine (mg)	0.291
Vitamin A (IU)	3131
Vitamin C (mg)	127.7
Vitamin E (mg)	1.58
Vitamin K (Âµg)	4.9
**Minerals**	
Sodium (mg)	4
Potassium (mg)	211
Calcium (mg)	7
Magnesium (mg)	12
Phosphorus (mg)	26

**Source:** USDA National Nutrient data base (2019) [28].

**Table 2:** Polyphenol and flavonoid content in bell pepper fruits.

Bioactive Compound	Bell Pepper Color				Ref.
Polyphenols	Green	Red	Yellow	Orange	
Total polyphenols (GAE mg/g)	4.51–52.65	7.86–42.57	7.44–43.59	12.35	[22,23,24,25,26,27]
3,4-Dihydroxybenzoic acid (µg/g)		0.40			[26]
3,4,5-methoxy-cinnamic acid (µg/g)	14.69	13.82	13.61		[23]
4-Aminobenzoic acid (µg/g)	22.09	21.34	50.19		[23]
α-coumaric acid (µg/g)	3.36	7.65	6.41		[23]
Benzoic acid (µg/g)	66.55	23.17–111.81	173.04		[23,26]
Catechol (µg/g)	279.42	89.77	225.73		[23]
Chlorogenic acid (µg/g)	60.84–290.08	60.47–221.53	103.78–136.51	117.54	[23,27]
Cinnamic acid (µg/g)	3.51	8.11	4.65		[23]
Gallic acid (µg/g)	89.98	115.74	119.48	900	[23,35]
Caffeic acid (µg/g)	18.09-108.82	41.33-67.78	52.42–62.96	38.03	[23,27]
Ellagic acid (µg/g)	106.67	172.18	144.52		[23]
Ferulic acid (µg/g)	23.59–48.42	11.88–27.67	24.75–35.14	13.45	[22,23]
Myricetin (µg/g)	658.19	244.33	151.35	100.62	[27]
P-Coumaric acid (µg/g)	19.62–46.69	9.96–26.07	18.14–24.75	13.45	[22,23]
P-OH-benzoic acid (µg/g)	65.85	395.16	123.19		[23]
Protocatechuic acid (µg/g)	116.09	97.21	95.37		[23]
Pyrogallol (µg/g)	572.77	757.66	2175.89		[23]
Resveratrol (µg/g)	174.34	111.57	90.78	89.72	[27]
Rosmarinic acid (µg/g)		120			[20]
Sinapinic acid (µg/g)		117			[20]
Vanillic acid (µg/g)	43.85	11–17.70	31.62		[23,26]
Vanillin (µg/g)		0.11			[26]
**Flavonoids**					
Total flavonoids (QE mg/g)	2.1–41	3.5–39	2.4–33	12.35	[24,27,34]
Apig. 6-arbinose 8-galactose (µg/g)	151.66	156.42	67.88		[23,24]
Apig. 6-rhamnose 8-glucose (µg/g)	170.96	314.70	77.31		[23]
Apigenin. 7-O-neohespiroside (µg/g)	33.55	40.27	4.51		[23]
Apegnin (µg/g)	2.12	36.28	1.54		[23]
Catechin (µg/g)	295.39	793.50	745.53	4.81	[23]
Epicatechin (µg/g)		505			[20]
Hespirtin (µg/g)	38.05	37.00	7.07		[23]
Hespirdin (µg/g)	1065.65	1513.13	213.06		[23]
Kampferol (µg/g)	22.48	31.15	9.53		[23]
Luteolin 7-glucose (µg/g)	181.12	413.57	92.21		[23]
Luteolin (µg/g dw)	62.31	68.43	95.89	56.34–154.03	[22,27]
Naringenin (µg/g)	13.64	1.54	2.12		[23]
Naringin (µg/g)	275.00	50.13	190.19		[23]
Quercetin (µg/g)	16.24–71.71	46.36–91.98	9.66–102.33	92	[22,23]
Quercetrin (µg/g)	394.23	9.97–241.83	62.34	42.87	[23,27,35]
Rutin (µg/g)	93.43	290.39	49.51		[23]

GAE: gallic acid equivalent: QE: quercetin equivalent.

**Table 3:** Carotenoids content in bell pepper fruits.

Carotenoids	Bell Pepper Color				Ref.
	Green	Red	Yellow	Orange	
**Total carotenoids (µg/g)**	1219–1513.5	7137–8800	2236.3–2834	5292	[23,26,27,42]
5,6,-epoxide capsanthin (µg/g)		513			[43]
α-carotene (µg/g)	3.56		4.22–21.27	9.02	[22,44]
β-carotene (µg/g)	1.86–12.2	0.70–43.9	3.86–15.9	56.6	[27,34,44]
13-Cis-β-carotene (µg/g)	10.7	36		12	[45]
9-Cis-β-carotene (µg/g)	12.9	38	3.2		[45]
9,13-Cis-β-carotene (µg/g)	11.6	139		12.5	[45]
β-Zea-carotene (µg/g)	97.3			4.6	[45]
α-cryptoxanthin (µg/g)	27	0.9–27		0.3	[34,45]
β-cryptoxanthin (µg/g)	4	40.49	7.55–19.5	19.45	[22,44]
Cis-β-cryptoxanthin (µg/g)	20	20	1.1	0.3	[44]
Antheraxanthin (µg/g)		44			[43]
Capsanthin (µg/g)	16.13	178.20	45.48	45.48	[22]
Capsorubin (µg/g)		1.4–48			[34,43]
Cis-beta-carotene (µg/g)	9.64	34.28	6.81	8.32	[22]
Cis-capsanthin		3.8			[43]
Cis-zexanthin (µg/g)		1.5			[34]
Chlorophyll (µg/g)	150.8	52.3	61.4		[24]
Cryptoxanthin (µg/g)		3.2			[34]
Cryptoflavin (µg/g)		2.1			[34]
Cucurbitaxanthin (µg/g)		81			[43]
Lycopene (µg/g)		4.8–322	2.5		[46,47]
Lutein (µg/g)	60.04–76.5		95.5–115.16	45.16	[22,44]
13-Cis-luteion	3	12			[45]
All-trans-lutein	14	37	58		[45]
Mutatoxanthin (µg/g)		49			[43]
Neoxanthin (µg/g)	190				[43]
Retinol (RE µg/g)	0.313		1.57		[44]
Trans-β-Carotene (µg/g)	13.09	41.72	6.81	8.32	[22]
Violaxanthin (µg/g)	12	48			[43]
Cis-violaxanthin (µg/g)	0.5	174	1.8		[45]
Zeaxanthin (µg/g)	35	8.8–70.71	48.3	191.76	[22,27,44]
Cis-zeaxanthin (µg/g)		24			[45]

**Table 4:** Antioxidant capacity by different methods for bell pepper.

Bell Pepper Color	Source	Bioactive Extracts or Compounds	Method of Antioxidant Capacity			Ref.
			ABTS• (µmol TE/g)	DPPH• (%)	FRAP (µg TE/g)	
Green	Fruit pulp	Ethanolic extract		78		[54]
		Ethanolic extract		40		[45]
		Methanolic extract	630	* 1153		[55]
		Methanolic extract		80	1400	[56]
		Ethanolic extract	17.17	^¥^ 2.28	^¥^ 3.99	[57]
		Ethanolic extract		^¥^ 25.15	30.15	[22]
		Methanolic extract		* 1114		[23]
		Methanolic extract		90		[58]
		NI	105	^¥^ 70		[27]
		Methanolic extract	4	^¥^ 30	46	[59]
	Fruit juice	Direct extraction	8.64	^¥^ 0.86		[60]
	Seeds	Ethanolic extract	89.25	^¥^ 11.32	^¥^ 9.94	[57]
		Aqueous extract		0.413		[51]
Red	Fruit pulp	Ethanolic extract		79.65		[54]
		Methanolic extract		54–70		[42]
		Ethanolic extract		80		[46]
		Lipophilic fraction	4.05			[61]
		Ethanolic extract		50		[45]
		Methanolic extract	800	* 882		[55]
		Methanolic extract		18		[35]
		Ethanolic extract		^¥^ 23.79	28.12	[22]
		Methanolic extract		* 1832		[23]
		Aqueous extract		* 366	^*^ 125	[26]
		Methanolic extract		80		[58]
		NI	90	^¥^ 80		[27]
		Methanolic extract	55.64	76		[62]
		Ethanolic extract		50		[63]
		Methanolic extract	4	^¥^ 25	39	[59]
		Methanolic extract		^¥^ 10		[64]
	Fruit juice	Direct extraction	14.02	^¥^ 1.05		[60]
Orange	Fruit pulp	Ethanolic extract		70		[54]
		Lipophilic fraction	5.20			[61]
		Ethanolic extract		75		[45]
		Methanolic extract	880	* 694		[55]
		Methanolic extract		21		[35]
		Ethanolic extract		^¥^ 22.20	25.20	[30]
		NI	85	^¥^ 50		[27]
	Fruit juice	Direct extraction	13.66	^¥^ 1.12		[60]
Yellow	Fruit pulp	Lipophilic fraction	3.33			[61]
		Ethanolic extract		64.90		[54]
		Ethanolic extract		70		[46]
		Ethanolic extract		80		[45]
		Methanolic extract	790	* 811		[55]
		Methanolic extract		22		[35]
		Aqueous ethanol extarct		^¥^ 18.23	19.87	[22]
		Methanolic extract		* 3267		[23]
		NI	65	^¥^ 60		[27]
		Methanolic extract	5	^¥^ 35	40	[59]
Purple	Fruit pulp	Methanolic extract		20		[35]
Dark violet	Fruit pulp	Methanolic extract		15		[35]

NI: no information; * IC_50_ = µg/mL; ^¥^ µmol TE/g.

**Table 5:** Antimicrobial activity of bell pepper extracts.

Bell Pepper Color	Source	Bioactive Extracts or Compounds	Dose	Model Assay	Effect	Ref.
NI	Fruit pulp	Isopropanol extract	20 µL of extract	DDA against *L. monocytogenes, S. typhymurium, B. cereus,* and *S. aureus*	Extract showed inhibition of growth in four bacteria.	[67]
Red	Fruit pulp	Methanolic extract	20 µg/mL	*DDA* against *B. cereus, E. coli, S. aureus,* and *P. aeruginosa*	Strain-dependent antimicrobial effect.	[68]
Yellow	Fruit pulp	Methanolic extract	20 mg/mL	*B. cereus, E. coli, S. aureus,* and *P. aeruginosa*	Strain-dependent antimicrobial effect.	[68]
Red	Fruit pulp	Ethanolic extract	300 mg/L	*F. andiyasi* and *Cochliobolus* spp.	Extracts showed fungistatic effect	[69]
Red	Fruit pulp	Ethanolic extract	5% *v*/*v*	*Cholletotrichum gloeosporioides*	Extracts showed antifungal effect.	[70]
NI	Fruit pulp	Ethanolic extract	1.5 mg/100 g	*S. typhimurium* and *P. aureginosa*	Extract showed bactericidal effect against pathogenic bacteria	[71]
NI	Fruit pulp	NI	1000 µL of crude extract	DDA against *Escherichia coli* O157H:7	Extract exhibited antimicrobial effect in a dose-dependent manner.	[72]
NI	Fruit pulp	NI		DDA against *S. aureus*, *L. monocytogenes*, *S. typhimurium*, *E. coli*, *B. subtilis*, *P. mirabilis, L. acidophilus, and L. plantarum*	Extract showed antimicrobial effect against pathogenic bacteria, while in lactic acid bacteria exhibited a prebiotic-like effect.	[73]
	Leaves	Peptide	NI	DDA against *Clavibacter michiganensis* spp. *michiganensis, Ralstonia solanacearum, Pseudomona syringae* pv. *tomato, Xanthomonas axonopodis* pv. *phaseoli, and Erwinia carotovora* sp. *carotovora*	Antimicrobial effect was seen in a strain- and concentration-dependent response.	[53]
	Leaves	Peptide	NI	DDA against *Clavibacter michiganensis* spp. *michiganensis and Ralstonia solanacearum*	Peptide exhibited an effect on microbial growth.	[52]

NI: no information; DDA: disk diffusion assay.

**Table 6:** Immunomodulatory effects of bell pepper extracts.

Color of Bell Pepper	Source	Bioactive Extracts or Compounds	Dose	Model Assay	Effect	Ref.
NI	NI	Nor-dihydrocapsiate	NI	Bioassay in Jurkat cells and BALB/c mice model	Compound exhibited immunosuppressive effects in a dose-dependent response via the inhibition of NF- kBis by phosphorylation of MAPK p38.	[74]
	Leaves	Aqueous extract	5 µg/mL	In vitro on mouse spleen cells	Aqueous extract had anti-inflammatory effects mediated by suppression of the T-cell activation.	[75]
Red	Pulp powder	Pectin polysaccharide isolated	40–100 mg/kg	In vivo in male BALB/c mice model	Extract decreased TNF release.	[76]
Red	Pulp powder	Aqueous extract	0.75 and 1.5 mg/mL	In vitro murine splenocytes and B cells	The extract promoted the production of IgM and IgG.	[77]
Red	Pulp powder	Aqueous extract	0.375, 0.75, 1.5, 2.25 mg/mL	In vitro and Ex vivo murine splenocytes and B cells	Aqueous extract promoted the production of both IgM and IgG antibodies in polyclonal response.	[78]
Red	Fruit pulp	Aqueous extract	1.5 mg/mL	In vitro and Ex vivo murine splenocytes and B cells	The extract dose-dependently increased polyclonal IgM production.	[79]

NI: no information.

**Table 7:** Effects of bell pepper extracts on diverse pathologies.

Pathology	Bell Pepper Color	Source	Bioactive Extracts or Compounds	Dose	Model Assay	Effect	Ref.
Diabetes	Green	Fruit juice	Whole juice/ethanol extracts	50 mg/mL	α-glucosidase inhibitory activity	Extract exhibited α-glucosidase inhibitory effects.	[80]
	Green	Fruit juice	Ethanol extracts	100 µg/mL	growth of 3T3-L1 preadipocytes	Extract increased the survive rate of preadipocyte cells	[80]
	Green	Fruit juice	Ethanol extracts	100 µg/mL	3T3-L1 differentiation into adipocytes induced	Extracts promote the 3T3-L1 cells differentiation rate	[80]
	NI	Fruit juice	Fruit juice	100 mL/twice a day	Randomized controlled study in humans	Fruit juice reduces post-prandial blood glucose and blood pressure.	[81]
	Red	Fruit pulp	Ethyl acetate extracts	20 µL	In vitro in HeLa cells	Extract inhibited the protein islet amyloid polypeptide.	[82]
	Red	Fruit pulp	Extract mixed with virgin olive oil	2 to 8 mL/kg body weight	In vivo animal assay in adult male rats	The mixture inhibited amylase and α-glucosidase activity.	[83]
	Green Red Yellow	NI	methanol extract	NI	NI	Extracts had α-glucosidase-inhibitory effects.	[84]
Cancer	NI	Powdered	Aqueous extracts	10% *v*/*w*	animal assay with *Drosophila* larval (SMART assay)	Aqueous extracts showed antimutagenic activity.	[85]
	Red	Fruit pulp	Methanol extract	125 µg/mL	In vitro in NIH3T3 and A549 cells	The extract exhibited strong cytotoxicity in A549 cells.	[68]
	Yellow	Fruit pulp	Methanol extract	125 µg/mL	In vitro in NIH3T3 and A549 cells	Selective cytotoxic activity against A549 cells.	[68]
	Green	Fruit pulp	Pectic polysaccharides	150 mg/kg	In vivo animal model in Ehrlich tumor-bearing mice	Significantly reduced tumor growth.	[49]
	Green	Fruit pulp	Pectic polysaccharides	0.1 mg/mL	In vitro in lineages of human mammary cancer cells (MCF-7, MDA-MB-231, and MDA-MB-436)	Selective cytotoxic activity against MCF-7, MDA-MB-231, and MDA-MB-436 cell lines.	[49]
	Green Yellow Red	Fruit pulp	Polyphenol mixtures	1.2 mg/L	In vitro in human gastric adenocarcinoma cells, A549 human lung carcinoma cells, and HeLa human cervical carcinoma cells	Extracts showed cytotoxic effects against all cancer cell lines in a dose-dependent response.	[86]
Alzheimer’s disease	NI	Powdered	Aqueous extracts / E-capsiate, Z-capsiate, dihydrocapsiate and nor-dihydrocapsiate,	1–10 g/L	In vitro Peptides agregation test	Bell pepper extracts were able to inhibit b- secretase activity and aggregation of Ab1–40 peptides	[87]

NI: no information.

**Table 8:** Applications of bell pepper extracts in food industry.

Application	Color	Source	Bioactive Extracts or Compounds	Dose	Model Assay	Effect	Ref.
Food preservation	NI	Fruit pulp	Ethanolic extract	1.5 mg/100 g	Raw beef meat	Extract help to extend the shelf life of beef meat.	[71]
	NI	Fruit pulp	NI	5% *v*/*v*	Grounded meat	Extract help to extend the shelf life of ground meat.	[72]
Natural colorant	Red	Fruit pulp	Ethanolic extract	NI	Yogurt	Capsules improve color of yogurt.	[89]
	Red	Fruit pulp	Ethanolic extract	10% *w*/*v*	Yogurt	Capsules improve sensory attributes of yogurt.	[20]
	Yellow	Fruit pulp	Ethanolic extract	0.6 g/L	Isotonic drink	Compounds improve the drink color.	[90]

NI: no information.

## Data Availability

Not applicable.
